# Transcutaneous Electrical Acupoint Stimulation Combined With Auricular Acupressure Reduces Postoperative Delirium Among Elderly Patients Following Major Abdominal Surgery: A Randomized Clinical Trial

**DOI:** 10.3389/fmed.2022.855296

**Published:** 2022-06-15

**Authors:** Qianqian Fan, Chong Lei, Yonghui Wang, Nannan Yu, Lini Wang, Jingwen Fu, Hailong Dong, Zhihong Lu, Lize Xiong

**Affiliations:** ^1^Department of Anesthesiology and Perioperative Medicine, Xijing Hospital, Fourth Military Medical University, Xi’an, China; ^2^Department of Traditional Chinese Medicine, Xijing Hospital, Fourth Military Medical University, Xi’an, China; ^3^Department of Anesthesiology and Perioperative Medicine, Translational Research Institute of Brain and Brain-Like Intelligence, Shanghai Fourth People’s Hospital Affiliated to Tongji University School of Medicine, Shanghai, China

**Keywords:** transcutaneous electric acupoint stimulation, auricular acupressure, elderly, abdominal surgery, delirium

## Abstract

**Background:**

Postoperative delirium is common in elderly patients following major surgery. This study aimed to assess the effect of transcutaneous electrical acupoint stimulation combined with auricular acupressure on the incidence of postoperative delirium among older patients undergoing major abdominal surgery.

**Methods:**

In this single-center, randomized controlled clinical trial, 210 patients aged 65 years or older undergoing major abdominal surgery were randomized to receive either intervention treatment (transcutaneous electrical acupoint stimulation started at 30 min before anesthesia until the end of the surgery, followed by intermittent auricular acupressure in the first three postoperative days; *n* = 105) or standard care (*n* = 105). The primary outcome was the incidence of delirium at the first seven postoperative days or until hospitalization depended on which came first. Secondary outcomes included delirium severity, opioid consumption, postoperative pain score, sleep quality, length of postoperative hospital stay, and postoperative 30-day complications. Enrollment was from April 2019 to March 2020, with follow-up ending in April 2020.

**Results:**

All of the 210 randomized patients [median age, 69.5 years, 142 (67.6%) male] completed the trial. The incidence of postoperative delirium was significantly reduced in patients received intervention treatment (19/105 (18.1%) vs. 8/105 (7.6%), difference, –10.5% [95% CI, –1.5% to –19.4%]; hazard ratio, 0.41 [95% CI, 0.18 to 0.95]; P= 0.023). Patients in the control group had a higher postoperative Memorial Delirium Assessment Scale (4 *vs.* 3; difference, –1; 95% CI, –1 to 0; *P* = 0.014) and a greater increase in Pittsburgh Sleep Quality Index score from baseline to postoperative day three (2.5 *vs.* 2.0; difference, –1; 95% CI, –2 to –1; *P* < 0.001) than patients in the intervention group. No significant difference was observed as of other secondary outcomes.

**Conclusion:**

In elderly patients undergoing major abdominal surgery, transcutaneous electrical acupoint stimulation combined with auricular acupressure reduced the incidence of postoperative in-hospital delirium compared with standard care. A multicenter, randomized clinical trial with a larger sample size is necessary to verify these findings.

**Clinical Trial Registration:**

[https://clinicaltrials.gov], identifier [NCT03726073].

## Introduction

Postoperative delirium is an acute neurological disorder that commonly occurs within the first three days after the operation (1, 2). Delirium prevalence ranges from 18 to 35% in general medical services and reaches 50% in elderly patients after high-risk surgery (3). It has been documented that postoperative delirium is associated with increased mortality, morbidity, and healthcare costs (4, 5). Despite efforts to decrease its occurrence, the incidence of postoperative delirium remains at 15%–54% in elderly patients after major abdominal surgery (6, 7).

The etiology of postoperative delirium is multifactorial and not fully elucidated. Cumulative evidence has shown that it may be associated with neuroinflammation, alteration in neurotransmitters, subclinical cerebral vascular events, and can be precipitated by factors such as pain, hypotension, and electroencephalogram suppression during surgery (1, 8). Acupuncture, a traditional Chinese medicine, has been reported to have anti-inflammatory and neuroprotective properties (9, 10). It also alleviates perioperative hypotension, reduces anesthetic and analgesic consumption, and improves sleep disorders (9, 11). Therefore, acupuncture and related techniques might exert a beneficial influence in reducing postoperative delirium.

Evidence supporting the use of acupuncture on delirium prevention is limited. A pilot study has shown that transcutaneous electrical acupoint stimulation applied on pre-and intraoperative has a trend of alleviating postoperative delirium in elderly patients with silent lacunar infarction undergoing spinal surgery (6.3% *vs*. 25.0%; relative risk, 0.25; 95% CI, 0.06 to 1.09) (12). Since the effect of a single session of electronic acupuncture only lasted for two or 3 h (9, 13), the transitory acupuncture protocol in the study may attribute to the limited effect of acupuncture on delirium prevention. Previous studies reported that delirium mainly occurs within postoperative three days (14, 15); thus, adding acupuncture interventions on postoperative three days may augment its preventive effects. Postoperative auricular acupuncture has shown its effect on relieving anxiety in post-cesarean section women (16) and preventing postoperative agitation in geriatric patients (17). In clinical practice, body and auricular acupuncture have been combined for synergistic effects, such as sleep improvement and opioid rescue (18, 19). However, the combined effects of body acupuncture and auricular acupuncture for delirium prevention in elderly patients have not been explored.

This study hypothesized that pre-and intra-operative transcutaneous electrical acupoint stimulation combined with auricular acupressure treatment for the first three postoperative days compared with standard care would reduce the incidence of postoperative delirium among elderly patients undergoing major abdominal surgery.

## Materials and Methods

### Study Design

This was a single-center, prospective, randomized, assessor-blinded trial. Ethical approval was obtained from the Institutional Review Board of Xijing Hospital (No. KY20182080-F-1) in Xi’an, Shaanxi Province of China. The trial was registered at ClinicalTrials.gov before patient enrollment (NCT03726073, October 31, 2018^1^). Written informed consent was obtained from all patients before randomization.

### Participants

Participants 65 years or older with American Society of Anesthesiologists physical status class ≤ III and scheduled for elective, major abdominal surgery (including hepatobiliary and pancreatic, urologic, gastrointestinal, or gynecological) were eligible for trial inclusion. Major surgery was defined by a planned more than two days hospitalization (20). Participants were required to complete a Mini-Mental State Examination and have a score higher than 20. Patients were excluded if they had a severe visual or auditory impairment, literacy deficits, mental illness, history of brain injury or neurosurgery, with a history of alcohol or drug abuse, history of acupuncture treatment, or contradicted to transcutaneous electrical acupoint stimulation or auricular acupressure (for example, planted with pacemakers, with skin lesions, or allergy to surface electrodes).

### Randomization and Blinding

Participants were recruited into the study from the electronic medical record system of our hospital by research staff. After giving consent and completing the baseline assessment, eligible participants were randomly assigned to either the intervention group (transcutaneous electrical acupoint stimulation combined with auricular acupressure) or the control group (standard care) in a 1:1 ratio using simple randomization. The random allocation sequence was generated by the statistician.

Study-group assignments were concealed in opaque envelopes and revealed by a research nurse upon patients’ arrival at the operation room. The investigators including the research coordinator, outcome assessors, data collectors, and the statistician were blinded to treatment allocation. The acupuncturist and participants were not blinded. The care team (surgeons, anesthesiologists, and nurses) were aware that an acupuncture study was undergoing but were blinded to study hypothesis and intervention protocol. Participants were informed not to discuss the group allocation or the aim of the study with investigators (except the acupuncturist) during the whole process of the study. Assessors would communicate with the ward nurses, and ask the nurses to let the patients wear an earmuff (loose enough to ensure patients could hear the assessors’ questions) before every postoperative evaluation. In addition, interactions between the assessors, participants and their family members were limited to the questions on the case report form. To assess the frequency of unblinding to group allocation, the assessors were asked to guess which treatment this patient had received after a patient was discharged from the hospital.

### Intervention

The acupressure protocol used in the present study was based on previous publications relating to acupoint selection in symptom management (12), investigators’ previous publications (9), and clinical experience of the acupuncturist in the team who is specializing in acupuncture clinical practice with seven years.

In the intervention group, participants received transcutaneous electrical acupoint stimulation through bilateral Hegu (LI4), Neiguan (PC6), and Zusanli (ST36) acupoints after entering the operation room. The neuroprotective effects and the exact locations of these three acupoints have been previously described (12, 21, 22). After entering the operation room, Hwato brand disposable electronic pads (size 50 mm × 50 mm) were placed on these acupoints after skin disinfection and then simultaneously connected to a transcutaneous electroacupuncture apparatus (SDZ-V, Suzhou Medical Appliance Company, Suzhou, Jiangsu, China). The transcutaneous electroacupuncture apparatus was set to provide a disperse-dense wave with an alternating frequency of 2/10 Hz. The current intensity was modulated between 5 and 20 mA (5 to 10 mA for the upper limbs, 10 to 20 mA for the lower limbs) and the final stimulus current of each acupoint was regulated individually until the De Qi sensation (a composite of sensations including soreness, numbness, distention, heaviness, and others such as coldness, warmness, and pain electric-shock feeling) was achieved at each acupoint. The electrostimulation was started from 30 min before anesthesia induction and up to the end of surgery.

Postoperatively, after arriving at the post-anesthesia care unit, patients in the intervention group were tapped *Vaccaria* seeds (Hebei Heshi Medical Apparatus and Instruments Co., LTD, Hengshui, China) at seven auricular points (Shenmen, Point Zero, Sympathetic, Subcortex, Heart, Liver, and Endocrine) located on the right ear by the acupuncturist. These auricular acupoints were previously used alone or combined to manage postoperative agitation and sleep disturbances (17, 23, 24). From postoperative day one to day three, participants were instructed to manually stimulate each auricular acupoint for 30 s, five times a day, for three days (From nine AM to nine PM, around every 3 h). Participants were instructed on the procedure, stimulation techniques, duration and intensity of auricular acupressure, the methods of keeping acupressure patches in the right place and protecting them, and were asked to document the time of auricular acupressure application and any side-effects in a diary sheet. The acupuncturist verified the quality of auricular acupressure daily. The pastes with seeds were removed after three days.

Participants in the standard care group did not receive any acupuncture intervention during the study period.

### Anesthesia Protocol

Anesthesia was induced with intravenous propofol (1–2 mg/kg) or etomidate (0.2–0.3 mg/kg), sufentanil (3 μg/kg), and rocuronium (1 mg/kg). After the loss of consciousness, an endotracheal tube was intubated. Anesthesia was maintained with sevoflurane inhalation (0.7–1.7 minimum alveolar concentration) and remifentanil infusion (0.05–0.2μg/kg/min). Rocuronium was administered as indicated (0.2 mg/kg). The depth of anesthesia was adjusted based on the hemodynamic indices and bispectral index (BIS). The BIS was maintained at 40–60 during surgery. Use of dexmedetomidine, midazolam, anticholinergic drugs, and haloperidol was avoided unless they were indicated for delirium rescue therapy. Standard monitoring procedures included electrocardiography, pulse oximetry, capnography, and inspiratory and expiratory sevoflurane concentrations. Invasive blood pressure monitoring was applied when necessary. A lung-protective ventilation strategy and multimodal analgesia were applied intraoperatively. Analgesia was assisted by intravenous administration of parecoxib sodium (40 mg) and oxycodone (0.1 mg/kg) preoperatively and local anesthetic infiltration in the surgical wound (0.5% ropivacaine) postoperatively. Postoperative intravenous patient-controlled analgesia was applied to patients during postoperative three days based on anesthesiologists’ clinical experience. Patient-controlled intravenous analgesia was established with 100 ml of 1 μg/ml sufentanil and 80 μg/ml butorphanol, programmed to deliver a background infusion of 0.015 ml/kg/h and 0.5 mL bolus with a lockout interval of 10 min. Parecoxib sodium (40 mg) was given intravenously every 12 h during the postoperative three days. An intravenous bolus of rescue oxycodone (0.05–0.1 mg/kg) was available every 6 h if the pain was intolerable.

### Outcomes

Two trained investigators who were not involved in the study intervention, anesthesia, and clinical care of patients performed outcome assessment. The primary outcome was the incidence of delirium during the first seven postoperative days or hospitalization if patients were discharged within seven days postoperatively. The diagnosis of delirium and its subtype were based on the Confusion Assessment Method or Confusion Assessment Method-intensive care unit for intubated patients, the Richmond Agitation Sedation Scale, reports from family members, and medical records review (5, 25, 26). Delirium was assessed daily at around 6 PM. The Confusion Assessment Method algorithm required the identification of both an acute onset and fluctuating course and inattention and either disorganized thinking or an impaired level of consciousness (25). As ward nurses and surgeons would assess patients’ mental status at least once daily and describe the results in their notes, medical records would be reviewed. For delirium patients in the ward, a mix-type delirium would be diagnosed if the patients manifested a fluctuation of mental status and behaviors during the day. Delirium patients in the intensive care unit were classified using the Richmond Agitation Sedation Scale score (RASS) (26): (1) hyperactive type, consistently positive RASS score remains at + 1 to + 4; (2) hypoactive type, consistently neutral or negative Richmond Agitation Sedation Scale remains at –3 to 0; (3) mixed type, alternative positive and negative.

Secondary outcomes included delirium severity measured by the Memorial Delirium Assessment Scale, intraoperative opioid consumption, pain intensity both at rest and at movement assessed by the Numeric Rating Scale at 24 h, 48 h, and 72 h after surgery, sleep quality within the first three postoperative days by the Pittsburgh Sleep Quality Index, length of postoperative hospitalization, the occurrence of non-delirium and complications within postoperative 30 days.

### Statistical Analysis

The incidence of postoperative delirium was reported to be up to 23.9% in elderly patients after major surgery (5, 7). We assumed that the incidence of postoperative delirium would be reduced from 23.9% in the control group to 8.9% in the intervention group based on the effect reported in a small randomized controlled trial (12). With significance set at 0.05 and a power of 80%, 95 patients in each group were required (PASS 15.0 software, NCSS, Kaysville, UT) to detect the differences. Considering a loss to follow-up rate of 10%, we planned to enroll 210 participants, with 105 in each group.

Analyses of primary and secondary outcomes were performed both in the intention-to-treat and per-protocol populations. We did not plan an interim analysis. We did not conduct the missing data imputation as there were no missing data for the primary outcome and less than 5% for all secondary outcomes.

Categorical variables are presented as frequencies or proportions and analyzed using the chi-squared test or Fisher exact test. Continuous variables are presented as mean (standard deviation) or median (interquartile range) depending on distribution (the Shapiro-Wilk test was used to assess normality) and analyzed with independent samples *t*-test or Mann–Whitney *U* test. The relative risk and 95% confidence interval (CI) are used to describe the differences in dichotomous outcomes. The difference (and 95% CI) between medians were calculated with the Hodges–Lehmann estimator.

The primary endpoint was analyzed using the chi-squared test. The cumulative postoperative delirium incidence was calculated with the Kaplan–Meier estimator, with differences between groups assessed using the log-rank test. The hazard ratio and 95% CI estimated with a Cox regression model were used to describe differences if the proportional assumption was not violated. The Cox regression model was adjusted for age and sex.

The Memorial Delirium Assessment Scale within seven postoperative days was compared using a generalized linear mixed-effect model, with results presented as mean and 95% CI. Treatment assignment, time, and interaction between treatment assignment and time effects were included as fixed effects. Participants were included as random effects. The model covariates were unadjusted.

Sensitivity analysis included analysis of the primary and secondary outcomes in the per-protocol populations. We performed *post hoc* subgroup analyses using a Cox proportional hazards model to investigate the intervention effects on the cumulative delirium incidence in specific subgroups, including sex (male *vs.* female), age (< 70 years old *vs.* ≥ 70 years old), the education level (≤ 9 years of schooling or no qualification *vs.* >9 years of schooling), surgery type (upper abdominal surgery *vs.* lower abdominal surgery), and history of operation. We tested for treatment effect heterogeneity across various subgroups and reported the corresponding *P* values for interaction.

Statistical analyses were performed using SPSS 24.0 (IBM Corp., Armonk NY). Two-tailed tests were used in all analyses, and *P* < 0.05 was considered statistically significant.

## Results

### Trial Population and Baseline Characteristics

**FIGURE 1:**
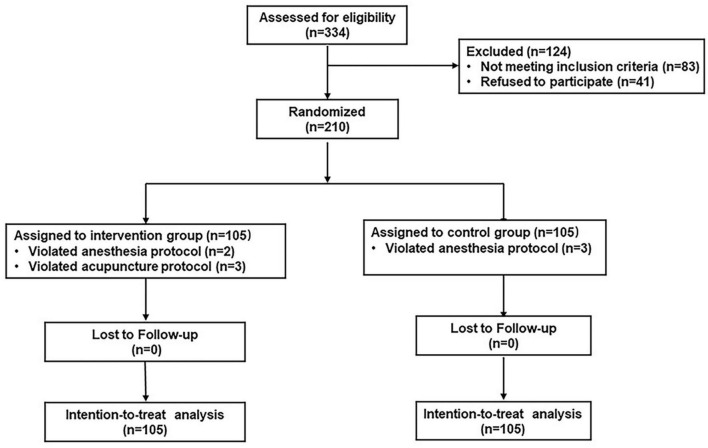
Trial profile. The intention-to-treat analysis included all randomized patients in the groups in which they were randomly assigned.

From April 17, 2019, to March 10, 2020, 334 patients scheduled for major abdominal surgery were screened for eligibility at Xijing Hospital, Xi’an, Shaanxi Province of China. Among them, 210 patients were enrolled and randomly assigned to either the intervention group (*n* = 105) or the standard care group (*n* = 105). Eight patients (two in the intervention group and three in the standard care group violated anesthesia protocol, three in the intervention group violated acupuncture protocol) were excluded from the per-protocol analysis (Figure 1).

Overall, most of the patients were male (142/210, 67.6%), and the median age was 69.5 (interquartile range, 67.0–73.0) years (Table 1). The types of surgery included hepatobiliary and pancreatic (52.4%), urologic (33.8%), gastrointestinal (10.5%), and gynecological (3.3%) procedures (Table 1). Baseline characteristics and surgical parameters did not differ between the two groups (Table 1). Forty-nine (23.3%) patients were discharged within postoperative six days, five (2.4%) patients (three in the control group and two in the intervention group) were transferred to the intensive care unit after surgery, and there were no differences between groups. The final visit of the last randomized patient took place on April 10, 2020.

**TABLE 1:** Baseline characteristics and surgical details of the intention-to-treat population.

Variables	Total (*n* = 210)	Control group (*n* = 105)	Intervention group (*n* = 105)
Age (years)	69.5	69.0	70.0
	(67.0–73.0)	(67.0-73.0)	(67.0-72.0)
**Sex**			
Male	142 (67.6)	70 (66.7)	72 (68.6)
Female	68 (33.4)	35 (33.3)	33 (31.4)
**ASA class**			
II	181 (86.2)	90 (85.7)	91 (86.7)
III	29 (13.8)	15(14,2)	14 (13.3)
Body mass index (kg/m^2^)	23.3 ± 2.8	22.7 ± 2.9	23.6 ± 2.9
Length of education (years)	9(5.8−12)	8(4.5−12)	9(6−12)
**Level of education**			
Illiteracy	14 (6.7)	9 (8.6)	5 (4.8)
Primary school	55 (26.2)	28 (26.7)	27 (25.7)
Middle school	59 (28.1)	31 (29.5)	29 (27.6)
High School	36 (17.1)	17 (16.2)	19 (18.1)
University	45 (21.4)	20 (19.0)	25 (23.8)
Mini-Mental State Examination score	27(25−29)	27(25−29)	28(25−29)
Carlson score	2(2−4)	2(2−4)	2(2−3)
Number of previous operations	1(0−2)	1(0−2)	1(0−2)
**Comorbidities**			
Hypertension	88 (41.9)	45 (42.9)	43 (41.0)
Coronary artery disease	23 (11.0)	12 (11.4)	11 (10.5)
Diabetes	37 (17.6)	23 (21.9)	14 (13.3)
Chronic bronchitis	9 (4.3)	5 (4.8)	4 (3.8)
Arrhythmia	24 (11.4)	10 (9.5)	14 (13.3)
Stroke	21 (10.0)	12 (11.4)	9 (8.6)
Current smoking	36 (17.1)	17 (16.2)	19 (18.1)
Alcoholism	6 (2.9)	4 (3.8)	2 (1.9)
**Surgical procedure**			
Hepatobiliary and pancreatic	110 (52.4)	54 (51.4)	56 (53.3)
Urologic	71 (33.8)	35 (33.3)	36 (34.3)
Gastrointestinal	22 (10.5)	11 (10.5)	11 (10.5)
Gynecological	7 (3.3)	5 (4.8)	2 (1.9)
Open surgery	121 (57.6)	56 (53.3)	65 (61.9)
Duration of anesthesia (min)	216 (165285)	227(170−285)	210(160−282)
Duration of surgery (min)	175(129−244)	175(132−239)	175(125−245)
Transferred to ICU after surgery	5 (2.4)	3 (2.9)	2 (1.9)
Discharged within postoperative six days	49 (23.3)	24 (22.9)	25 (23.8)
Number of patients using PCIA	156 (74.3)	77 (73.3)	79 (75.2)

*Data are presented as number (%), mean (SD), or median (interquartile range). Differences between groups were compared using the chi-squared, Mann–Whitney U, or Fisher’s exact test as appropriate. ASA: American Society of Anesthesiologists. ICU: Intensive care unit. PCIA: Patient-controlled intravenous analgesia.*

### Primary and Secondary Outcomes

**FIGURE 2:**
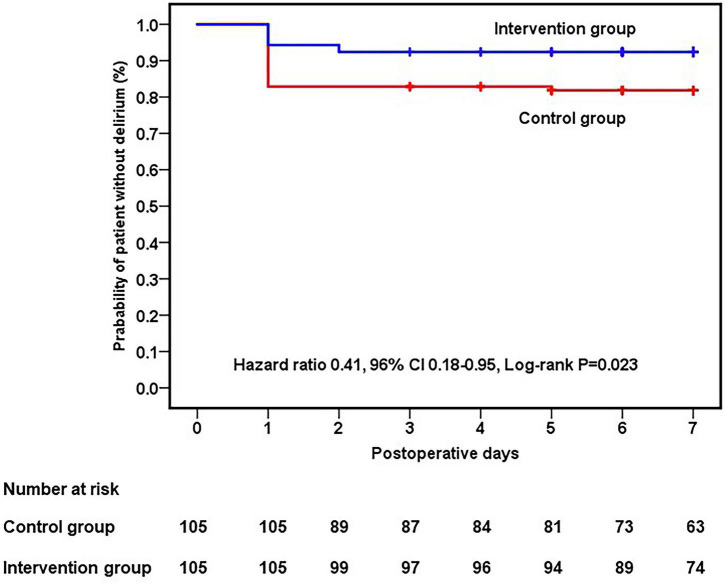
Kaplan–Meier curve showing the probability of a patient being delirium-free within seven postoperative days.

In the intention-to-treat population, the incidence of postoperative delirium during postoperative seven days was 7.6% (8/105) in the intervention group and 18.1% (19/105) in the control group (difference, −10.5% [95% CI, −1.5% to −19.4%]; relative risk, 0.42 [95% CI, 0.19 to 0.92]; *P* = 0.023; number needed to treat = 10 [95% CI, 5 to 64]; Table 2). The proportion of postoperative delirium-free patients significantly differed between the two groups (hazard ratio, 0.41; 95% CI, 0.18 to 0.95; *P* = 0.023) (Figure 2).

**TABLE 2:** Effectiveness outcomes analyzed in the intention-to-treat population.

Variables	Control group (*n* = 105)	Intervention group (*n* = 105)	Relative risk or difference (95% CI)	*P* value
**Primary outcome**				
^1^Overall incidence of delirium	19 (18.1)	8 (7.6)	0.42 (0.19 to 0.92)	0.023
**Secondary outcomes**				
Memorial Delirium Assessment Scale	4 (2–6)	3 (2–5)	−1 (−1 to 0)	0.010
The motoric subtype of delirium				0.025
Hypoactive	9 (8.6)	6 (5.7)		
Hyperactive	7 (6.7)	0 (0)		
Mixed	3 (2.9)	2 (1.9)		
Delirium on the day of surgery	16 (15.2)	6 (5.7)	0.38 (0.15 to 0.92)	0.024
^2^Delirium duration	3.0 (2.0–3.0)	2.0 (1.3–3.0)	0 (−1 to 1)	0.449
Intraoperative consumption of sufentanil (ng/kg/min)	2.4 (1.7–3.4)	2.4 (1.8–3.0)	−0.07 (−0.37 to 0.20)	0.642
Intraoperative consumption of remifentanil (μg/kg/min)	0.13 (0.11–0.14)	0.12 (0.10–0.14)	-0.01 (−0.01 to 0)	0.136
**Pain score at rest**				
Postoperative day 1	0 (0–1)	0 (0–2)	0 (0 to 0)	0.248
Postoperative day 2	0 (0–1)	0 (0–1)	0 (0 to 0)	0.679
Postoperative day 3	0 (0–1)	0 (0–1)	0 (0 to 0)	0.901
**Pain score with movement**				
Postoperative day 1	3(1–4)	3 (2–4)	1 (0 to 1)	0.290
Postoperative day 2	2 (1–4)	3 (1–4)	0 (0 to 1)	0.137
Postoperative day 3	2 (1–4)	3 (1–4)	0 (0 to 1)	0.390
**PCIA Drug Consumption (ml)**				
Postoperative 0–24 h	21.5 (18.2–25.8)	22.4 (16.5–25.1)	0.2 (–2.2 to 2.1)	0.859
Postoperative 24–48 h	20.8 (8.3–24.3)	21.5 (7.1–25.5)	0.1 (–2.5 to 2.9)	0.938
Postoperative 48–72 h	6.4 (0–22.5)	3.2 (0–21.6)	0 (–2.1 to 0)	0.302
**Pittsburgh Sleep Quality Index**				
Baseline	7 (5–10)	7(4.5–10)	0 (−1 to 1)	0.567
During postoperative three days	11 (8–14)	8 (6–13)	−2 (−3 to -1)	0.001
Changes from baseline	2.5 (2.0–4.8)	2.0 (1.0–3.0)	−1 (−2 to -1)	< 0.001
Length of postoperative hospitalization (days)	8 (7–10)	8 (7–10)	0 (−1 to 1)	0.988
^3^ Incidence of non-delirium complications	21 (20.0)	12 (11.4)	0.57 (0.30 to 1.10)	0.088

*Data are presented as number (%) or median (interquartile range). Differences between groups were compared using the chi-squared, Mann–Whitney U, Fisher’s exact test, or generalized linear mixed effect models as appropriate. ^1^Occurrence of delirium at any time during the first seven postoperative days or hospitalization if the patient was discharged within seven postoperative days. ^2^Delirium duration was calculated only for patients who experienced delirium. ^3^Occurrence of any non-delirium complication within 30 days postoperatively. PCIA: Patient-controlled intravenous analgesia. PCIA therapy was applied to seventy-seven patients in the control group and seventy-nine patients in the intervention group.*

**FIGURE 3:**
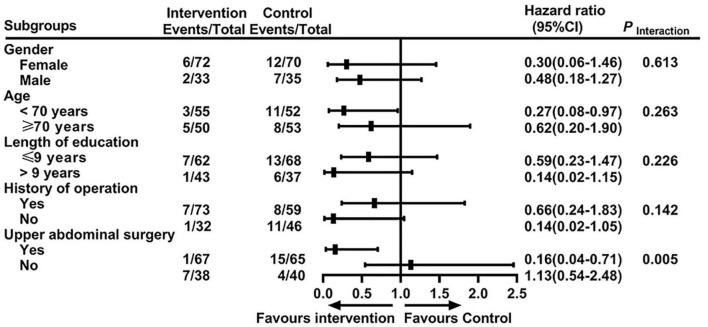
Forest plot of the subgroup analysis for the primary outcome.

Subgroup analyses using the Cox model showed that the interaction between surgery sites and treatment was statistically significant (*P* = 0.005, Figure 3). Patients who underwent upper abdominal surgery benefited more from the intervention than those who underwent lower abdominal surgery (hazard ratio, 0.16; 95% CI, 0.04 to 0.71).

**FIGURE 4:**
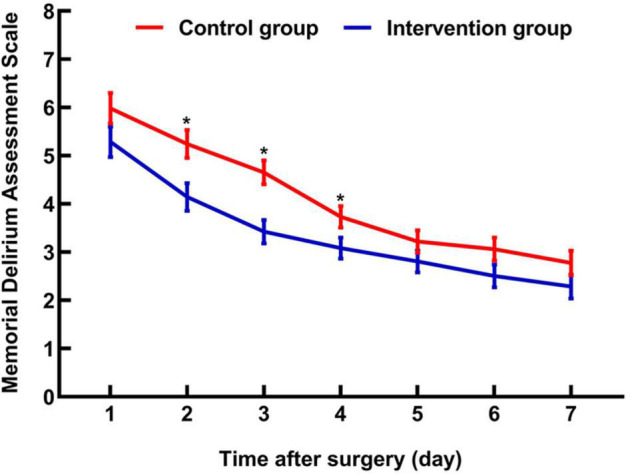
Changes in Memorial Delirium Assessment Scale during the first postoperative seven days. Makers indicate means, and error bars indicate standard errors. *Significant difference between groups.

The incidence of delirium on the day of surgery was 5.7% (6/105) in the intervention group and 15.2% (16/105) in the control group (relative risk, 0.38; 95% CI, 0.15 to 0.92; *P* = 0.024). Delirium severity measured by Memorial Delirium Assessment Scale was lower in the intervention group than in the control group (3 *vs.* 4; difference, –1; 95% CI,–1 to 0; *P* = 0.010), with significant differences on postoperative day two (*P* = 0.007), day three (*P* < 0.001) and day four (*P* = 0.037) (Figure 4). The control group had a significantly greater increase regarding Pittsburgh Sleep Quality Index score change from baseline to postoperative day three than the intervention group (2.5 *vs.* 2.0; difference, –1; 95% CI, –2 to –1; *P* < 0.001). The two groups did not differ significantly with respect to the duration of delirium, the intraoperative consumption of sufentanil and remifentanil, the Numeric Pain Rating Scale at rest and movement at postoperative 24 h, 48 h, and 72 h, the patient-controlled intravenous analgesia drug consumption during postoperative 0–24 h, 24–48 h, and 48–72 h, and the length of postoperative hospitalization (Table 2).

The per-protocol analysis yielded similar results (Supplementary Table 1).

### Adverse Events

Thirty-three surgery-related adverse events (12 in the intervention group and 21 in the control group) were documented within 30 days postoperatively. Adverse events (anastomotic leakage or bleeding, pneumonia, delayed feeding, readmission, sepsis, re-operation, death, acute kidney failure, new arrhythmia, and ileus), whether viewed individually (*P* for each > 0.20) or collectively (11.4% *vs.* 20.0%; relative risk, 0.57; 95% CI, 0.30 to 1.10; *P* = 0.088), did not significantly differ between the two groups (Table 3). Two patients in the control group died of postoperative septic shock, but no difference was found in all-cause 30-day mortality between groups.

**TABLE 3:** Postoperative complications within 30 days analyzed in the intention-to-treat population.

Variables	Control group (*n* = 105)	Intervention group (*n* = 105)	*P value*
Anastomotic leakage or bleeding	3 (2.9)	1 (1.0)	0.621
Pneumonia	3 (2.9)	3 (2.9)	1.000
Delayed feeding	2 (1.9)	1 (1.0)	1.000
Readmission	5 (4.8)	1 (1.0)	0.212
Sepsis	2 (1.9)	0 (0)	0.498
Re-operation	2 (1.9)	3 (2.9)	0.681
Death	2 (1.9)	0 (0)	0.498
Acute kidney failure	1 (1.0)	1 (1.0)	1.000
New arrhythmia	1 (1.0)	1 (1.0)	1.000
Ileus	0 (0)	1 (1.0)	1.000

*Data are presented as number (%). Differences between groups were compared using Fisher’s exact test.*

Skin temporary pain or paranesthesia was considered an expected adverse event with transcutaneous electrical acupoint stimulation and auricular acupressure treatment. One patient in the intervention group reported mild leg numbness, which was alleviated within three days. There was no withdrawal from adverse events.

### The Effectiveness of Assessor-Blinded

Outcome assessors were able to get a 54.3% rate for correctly guessing patients’ received therapy (59.0% for the intervention group and 49.5% for the control group). Outcome assessors’ perception of patients’ accepted treatment did not affect the primary outcome (Supplementary Table 2).

## Discussion

In this single-center, prospective, randomized trial, transcutaneous electrical acupoint stimulation combined with auricular acupressure reduced the incidence and severity of postoperative delirium and improved postoperative sleep quality in elderly patients undergoing major abdominal surgery.

Postoperative delirium developed in 18.1% of patients in the standard care group. This was in line with previous studies for non-cardiac surgeries, which range from 13 to 50% (3), but slightly lower than the reported rate that we used to estimate sample size (23.9%) (5). There are several reasons for this. First, compared with those in the previous study (5), patients in the current study were younger (median age: 69.5 years *vs.* 77.0 years) and higher delirium risk patients (such as American Society of Anesthesiologists physical status class > III and Mini-Mental State Examination score ≤ 20) were excluded. Second, anesthesia management was different between the two studies. Delirium risk factors such as midazolam, anticholinergic drugs, and haloperidol were avoided, and Bis-guided anesthesia management was applied during surgery in the present study. These reasons may explain the slightly lower incidence of delirium in this trial (1, 3).

A trend of reduced incidence of postoperative delirium (6.3% *vs.* 25%; relative risk, 0.25; 95% CI, 0.06 to 1.09) was reported when transcutaneous electrical acupoint stimulation was applied pre- and intraoperatively to 64 geriatric patients with silent lacunar infarction undergoing spine surgery (12). This was consistent with the delirium-prevention effect observed in our study (relative risk, 0.42; 95% CI, 0.19 to 0.92). The mechanisms of the delirium-sparing effect produced by transcutaneous electrical acupoint stimulation and auricular acupressure remain undetermined. Gao et al. demonstrated that transcutaneous electrical acupoint stimulation at bilateral LI4 and PC6 reduced neuroinflammation by lowering the permeability of the blood-brain barrier (12). Studies reported that one potential mechanism by which auricular acupressure may exert its anti-delirium effect is activating the locus coeruleus noradrenergic system, which is one of the central vagal relay centers and plays a critical role in the generating and regulating of delirium (27, 28). By stimulating the ear branch of the vagus nerve, auricular acupressure may exert an add-on neuroprotective effect by direct and indirect modulation of the activity and connectivity of the locus coeruleus noradrenergic system, thus modulating the release and uptake of noradrenaline and dopamine in some key brain regions, including the prefrontal cortex and hippocampus, which are postulated to be associated with attention, memory, and other cognitive dysfunction (27–29). As postoperative delirium has been speculated to be a harbinger for postoperative cognitive dysfunction, the mechanism of acupuncture to prevent postoperative delirium may be similar to those of acupuncture to prevent postoperative cognitive dysfunction, which is by attenuating systemic inflammation and neuroinflammation, reducing oxidative stress levels, improving synaptic plasticity, and reducing neuronal injury (10, 12, 30).

Subgroup analysis of our data suggested that patients who underwent upper abdominal surgery benefitted more from the intervention than patients who underwent lower abdominal surgery. It is unclear why this occurred, but this heterogeneity may be related to the acupoints we selected and the individual response differences toward acupuncture treatment. Kim et al. suggested that individual differences in acupuncture analgesia are associated with inherited genetic factors, adenosine monophosphate-activated protein kinase expression in the hypothalamus, spinal levels of neurotransmitters and pro-inflammatory cytokines, and the density of cholecystokinin receptors (31). The difference of acupuncture’s delirium-prevention effects between upper and lower abdominal surgery may share similar mechanisms and warrant further study. Our results also raise the question of whether the specificity of needling sites is essential to the therapeutic benefits of acupuncture.

Psychological distress, pain, and insomnia are often intertwined (32). Pain and sleep disorders are possible confounders of delirium severity, and thus attenuated the extent of postoperative pain and improved sleep quality might reduce the incidence of postoperative delirium (1, 33). Inconsistent with the previous studies (34, 35), acupuncture did not decrease intraoperative opioid consumption and postoperative pain in this trial. This may be related to the high opioid doses and a multi-model analgesia protocol adopted in the present trial.

The change from baseline to postoperative day three in the Pittsburgh Sleep Quality Index score was more significant in the control group than in the intervention group (2.5 *vs.* 2.0; between-group difference, –1; 95% CI, –2 to –1; *P* < 0.001), indicating that the acupuncture intervention in the present study has a beneficial effect on postoperative sleep quality. The improved sleep quality within the first three postoperative days in the present study may be attributed to auricular acupressure, as indicated in the previous studies that auricular acupoint stimulation improves sleep quality (36). The rationale of the auricular acupoints selected in this trial was based on their effects of calming the mind, relieving anxiety, and improving sleep quality (23). Hou et al. showed that auricular acupressure normalized disturbed sleep patterns and improved sleep quality via regulating the neuroendocrine system, neuroimmunological factors, neuroinflammation, and neural reflex, as well as antioxidation (37). However, whether auricular acupressure reduces postoperative delirium directly or indirectly by improving postoperative sleep quality is unclear, which needs to be elucidated in future studies.

The Confusion Assessment Method and Confusion Assessment Method-intensive care unit were used in this study to diagnose delirium as they are commonly used for postoperative delirium identification and easy to learn for non-psychiatrists, with high sensitivity (94% to 100%) and high specificity (90% to 95%) (25). As each assessment may take 20 min to 30 min, and assessments were conducted for postoperative seven days, a once-daily assessment was chosen in this study to improve participants’ compliance. However, it is possible that short periods of fluctuating mental status, inattention, disorganized thinking, or altered consciousness level may not be detected at the time of assessment. Additional medical records review was used in this study to minimize the risk of delirium misclassification, which has been accepted in previous studies (5, 38). Further research should add other screening methods such as the Consortium to Establish a Registry of Alzheimer’s Disease (39) and the Montreal Cognitive Assessment tests (40) to assess other diverse cognitive domains and verify the robustness of anti-delirium effects exerted from electrical acupoint stimulation combined with auricular acupressure.

This study has several limitations. First, this is a single-center study, while internal validity is not impacted, the external validity and generalizability of the results may be affected by clinical practice and therapeutic measures in various medical centers. The sample size is relatively small, the power of this intervention should be tested in a clinical trial with a larger sample size. Second, the enrolled patients and the acupuncturist were not blinded. Thus, an inherent risk of the Hawthorne effect cannot be ruled out in this study. The outcome assessors, data collectors and the statistician were blinded to reduce the risk of bias. Third, a sham acupuncture group was not used to eliminate the placebo effect. Future studies are needed to distinguish the specific and non-specific effects of acupuncture intervention for postoperative delirium. Fourth, the subgroup analysis was *post hoc*. Therefore, these findings should be interpreted as exploratory. In addition, the number of events in most subgroups was relatively small, which may limit the power to detect the difference among subgroups. Fifth, the dosage of postoperative rescue analgesic drugs was not recorded. However, perioperative pain management was strictly controlled, postoperative pain scores were lower, and the analgesic effect was comparable between the two groups. Standardized perioperative pain management and preservation of randomization would be expected to minimize the confounding from rescue drug consumption differences. Furthermore, although anesthesia management was conducted under the guidance of the BIS, the BIS and frequencies of burst suppression during surgery were not recorded and compared, potentially confounding and biasing the results (41).

Acupuncture is an important supplementary strategy for perioperative management (9), applications of acupuncture before, during, and after surgery as an adjuvant therapy will have a great clinical application. Future acupuncture studies should investigate how to best implement acupuncture in real-world clinical settings, such as setting up an acupuncture protocol and selecting the appropriate acupoints for specific surgeries and populations. To investigate the mechanisms of how acupuncture prevents postoperative delirium could provide essential insights in preventing postoperative delirium. As postoperative delirium and postoperative cognitive dysfunction share risk factors (30), a long-term follow-up to investigate the preventive effects of acupuncture intervention on postoperative cognitive dysfunction should also be included in future studies.

In conclusion, in this single-center, prospective, randomized study, transcutaneous electrical acupoint stimulation combined with auricular acupressure reduced the incidence of postoperative delirium in elderly patients undergoing major abdominal surgery. A multicenter randomized clinical trial with a larger sample size is necessary to verify these findings.

## Data Availability Statement

The raw data supporting the conclusions of this article will be made available by the authors, without undue reservation.

## Ethics Statement

The studies involving human participants were reviewed and approved by the Institutional Review Board of Xijing Hospital (No. KY20182080-F-1) in Xi’an, Shaanxi Province of China. The patients/participants provided their written informed consent to participate in this study.

## Author Contributions

QF, CL, ZL, HD, and LX contributed to the concept and design of the study and data interpretation. QF and ZL performed the study registration. QF and JF performed the screen of patients. NY performed the acupuncture intervention. YW and LW performed the outcome assessment and data collection. CL performed the data analysis. QF, CL, ZL, and HD prepared the primary manuscript. All the authors read and approved the final version of the manuscript.

## Conflict of Interest

The authors declare that the research was conducted in the absence of any commercial or financial relationships that could be construed as a potential conflict of interest.

## Publisher’s Note

All claims expressed in this article are solely those of the authors and do not necessarily represent those of their affiliated organizations, or those of the publisher, the editors and the reviewers. Any product that may be evaluated in this article, or claim that may be made by its manufacturer, is not guaranteed or endorsed by the publisher.

## Footnotes

1
https://clinicaltrials.gov/ct2/show/NCT03726073
