# Supplementary material for: Transcutaneous Electrical Acupoint Stimulation Combined With Auricular Acupressure Reduces Postoperative Delirium Among Elderly Patients Following Major Abdominal Surgery: A Randomized Clinical Trial
Source: Front Med (Lausanne). 2022 Jun 15;9:855296. doi: 10.3389/fmed.2022.855296 (PMC9240658; doi:10.3389/fmed.2022.855296)
Supplement: Supplementary file 1 [file Table_1.DOCX]

Supplementary Material

# Supplementary Table 1. Clinical outcomes according to the per-protocol analysis.

| Variables | Control group (n=102) | Intervention group (n=100) | Relative risk, or difference (95% CI) | *P* value |
| --- | --- | --- | --- | --- |
| **Primary outcome** |  |  |  |  |
| ^1^Overall incidence of delirium | 18 (17.6) | 8 (8.0) | 0.45 (0.21 to 0.99) | 0.041 |
| **Secondary outcomes** |  |  |  |  |
| Memorial Delirium Assessment Scale | 4 (2–6) | 3 (2–5) | -1(-1 to 0) | 0.014 |
| The motoric subtype of delirium |  |  |  | 0.035 |
| Hypoactive | 8 (7.8) | 6 (6.0) |  |  |
| Hyperactive | 7 (6.9) | 0 (0) |  |  |
| Mixed | 3 (2.9) | 2 (2.0) |  |  |
| Intraoperative consumption of sufentanil (ng·kg^-1^·min^-1^) | 2.4(1.7–3.4) | 2.4(1.8–3.3) | -0.01(-0.3 to 0.2) | 0.691 |
| Intraoperative consumption of remifentanil (μg·kg^-1^·min^-1^) | 0.13(0.10–0.14) | 0.12(0.11–0.14) | -0.01(-0.01 to 0) | 0.254 |
| Pain score at rest |  |  |  |  |
| Postoperative day 1 | 0 (0–1) | 0 (0–2) | 0 (0 to 0) | 0.329 |
| Postoperative day 2 | 0 (0–1) | 0 (0–1) | 0 (0 to 0) | 0.768 |
| Postoperative day 3 | 0 (0–1) | 0 (0–1) | 0 (0 to 0) | 0.823 |
| Pain score with movement |  |  |  |  |
| Postoperative day 1 | 3 (1–4) | 3 (2–4) | 0 (0 to 1) | 0.378 |
| Postoperative day 2 | 2 (1–4) | 3 (1–4) | 0 (0 to 1) | 0.179 |
| Postoperative day 3 | 2 (1–4) | 3 (1–4) | 0 (0 to 1) | 0.460 |
| Pittsburgh Sleep Quality Index |  |  |  |  |
| Baseline | 7.0 (5.0–11.0) | 7(4.0–10.0) | 0 (-1 to 1) | 0.565 |
| During postoperative three days | 11.0 (8.0–14.0) | 8.6(6.0–13.2) | -2 (-3 to -1) | 0.001 |
| Changes from baseline | 2.5 (2.0–5.0) | 2.0 (0–3.0) | -1 (-2 to -1) | <0.001 |
| Length of postoperative hospitalization (days) | 8 (7–10) | 8 (7–10) | 0 (-1 to 1) | 0.966 |
| ^2^Incidence of non-delirium complications | 21 (10.6) | 12 (12.0) | 0.58(0.30 to 1.12) | 0.099 |

Data are presented as number (%) or median (interquartile range). Differences between groups were compared using the chi-squared, Mann–Whitney *U*, Fisher's exact test, or a generalized linear mixed effect model. ^1^Occurrence of delirium at any time during the first seven days after surgery or hospitalization if the patient was discharged within postoperative seven days. ^2^Occurrence of any non-delirium complications within 30 days after surgery.

**Supplementary Table 2.** Primary outcome depending on therapy guess.

| Group | Guess | n | Number of Delirium | *P* value |
| --- | --- | --- | --- | --- |
| Intervention group | Intervention group | 62 | 4 | 0.714 |
|  | Standard care group | 43 | 4 |  |
| Standard care group | Intervention group | 53 | 8 | 0.420 |
|  | Standard care group | 52 | 11 |  |
